# Complementary therapies in early breast cancer: Oncologists’ evidence-based decisions in a Southeast European vignette survey

**DOI:** 10.17305/bb.2025.13413

**Published:** 2025-12-15

**Authors:** Teo Buhovac, Žarko Bajić, Mario Nalbani, Lazar Popović, Milan Risteski, Nikola Milašević, George Dimitrov, Anes Pašić, Marija Karaga, Eduard Vrdoljak

**Affiliations:** 1 Oncology Clinic, University Clinical Hospital Mostar, Faculty of Medicine, University of Mostar, Mostar, Bosnia and Herzegovina; 2 Research Unit “Dr. Mirko Grmek”, University Psychiatric Hospital “Sveti Ivan”, Zagreb, Croatia; 3 Biometrika Healthcare Research, Zagreb, Croatia; 4 Department of Oncology, General Hospital Dubrovnik, Dubrovnik, Croatia; 5 Oncology Institute of Vojvodina, Faculty of Medicine, University of Novi Sad, Novi Sad, Serbia; 6 Zan Mitrev Clinic, Skopje, North Macedonia; 7 Institute for Oncology, Clinical Centre of Montenegro, Podgorica, Montenegro; 8 Department of Medical Oncology, University Hospital “Tsaritsa Yoanna – ISUL”, Medical University of Sofia, Sofia, Bulgaria; 9 UPMC Hillman Cancer Centre Croatia, Bračak, Croatia; 10 Clinical Hospital Centre Split, School of Medicine, University of Split, Split, Croatia

**Keywords:** Integrative medicine, integrative oncology, breast neoplasms, complementary therapies

## Abstract

Complementary therapies are increasingly integrated into the framework of integrative oncology. While numerous complementary therapies provide potential benefits, some may also carry risks, including interactions with conventional cancer treatments. The degree to which oncologists’ real-world decisions regarding complementary therapies align with evidence-based guidelines remains uncertain. This study aimed to evaluate oncologists’ evidence-based decisions on whether specific complementary therapies should be prohibited, permitted, or recommended for early breast cancer treatment. We conducted a cross-sectional online survey that included a randomized vignette experiment involving oncology specialists and residents from seven Southeast European countries. The primary outcome was the percentage of accurate classifications of 28 therapy-indication pairs in neoadjuvant (NA) and adjuvant (ADJ) settings, benchmarked against published evidence. Correctness was assessed using both a strict definition (one correct option) and an expanded definition (accepting “allow” or “recommend” when supported by evidence). A total of 136 respondents met the inclusion criteria and provided paired responses. Median accuracy was found to be 52% (95% CI 48–55) under the strict definition and 70% (95% CI 67–72) under the expanded definition, with no significant differences observed between NA and ADJ settings. Evidence-based therapies, such as physical exercise and cognitive behavioral therapy, were most frequently recommended, whereas most other therapies received endorsement from fewer than 25% of respondents. Overall, oncologists exhibited moderate alignment with evidence, demonstrating a tendency to permit rather than actively recommend complementary therapies, even when evidence indicated potential benefits and safety. These findings underscore the necessity for targeted educational interventions aimed at enhancing oncologists’ understanding and ensuring the safe and informed integration of complementary therapies into clinical practice.

## Introduction

Complementary therapies encompass medical products and practices utilized in conjunction with, but never as a replacement for, standard cancer care. In this article, we define complementary therapies as treatments supported by evidence of safety and some proof of efficacy for specific indications. These therapies can be categorized into nutritional (e.g., diets, supplements, herbs, and probiotics), psychological (e.g., meditation, hypnosis, music therapy, and relaxation techniques), physical (e.g., acupuncture, massage, and spinal manipulation), or combined approaches (e.g., yoga and tai chi). Some therapies initially classified as complementary may eventually gain acceptance as part of standard medical care when supported by robust evidence.

Integrative medicine merges conventional and complementary therapies, respecting patient preferences and emphasizing their mental, physical, and spiritual well-being [1]. Over the past few decades, interest in and utilization of complementary therapies among cancer patients have markedly increased [2]. A multicenter European survey conducted across 14 countries found that 35.9% of cancer patients reported using some form of complementary therapies, with significant variation depending on the country [3]. Recently, cancer patients have increasingly turned to complementary therapies for symptom management and supportive care, rather than as alternatives to conventional cancer treatments, indicating a shift in patient attitudes and preferences [4]. In response to accumulating evidence and guidelines [5, 6], and recognizing the substantial symptom burden and side effects associated with conventional treatments, leading oncology institutions such as Memorial Sloan Kettering and MD Anderson have established integrative oncology programs and developed educational resources for patients and healthcare providers [7]. In 2002, the World Health Organization recommended that all nations formulate national policies and procedures regarding complementary therapies [8].

Given the high prevalence of complementary therapy use and associated risks, oncologists must be well-informed and proactive in discussing these therapies with their patients. Research indicates that over 50% of cancer patients believe their physicians should be able to address these therapies and consider integrating them into cancer care [9]. It is important to note that certain complementary therapies may adversely interact with oncology treatments [10–12]. Additionally, potential interactions with treatments for comorbidities must be considered, an often-underestimated issue [13]. Conversely, evidence suggests that complementary therapies can alleviate disease symptoms, reduce side effects of standard treatments, and enhance the quality of life for cancer patients [14–18]. Furthermore, there is growing evidence supporting the significance of physical activity and nutrition in maintaining the health of cancer patients, which has been associated with improved treatment outcomes [19–21].

Many studies reveal a disconnect between healthcare professionals’ limited knowledge of complementary therapies and their interest in acquiring further information [22]. Negative or uncertain attitudes among physicians toward complementary therapies, as perceived by patients, can heighten patient anxiety regarding potential treatment interactions [23]. Physicians often express skepticism about complementary therapies due to a perceived lack of scientific evidence supporting their efficacy [24]. This skepticism contributes to a passive approach, with many healthcare providers not routinely inquiring about patients’ use of complementary therapies [25]. Effective physician-patient communication is critical for building trust; improved communication can enhance the likelihood that patients will adhere to medical recommendations and may help mitigate harmful interactions between complementary and conventional therapies [26].

Recent advancements in breast cancer (BC) treatment, for both early-stage and metastatic disease, have significantly improved survival rates and expanded therapeutic options [27–30]. However, the introduction of novel systemic therapies also presents new challenges for clinicians, including managing treatment-related toxicities, maintaining quality of life, and navigating complex decision-making in clinical practice. These developments underscore the necessity for oncologists to understand how to safely incorporate integrative approaches into patient care. Therefore, monitoring and integrating evidence-based complementary interventions alongside evolving conventional treatments is increasingly vital for optimizing clinical outcomes and patient-centered care.

Globally, substantial progress has been made in incorporating complementary therapies into healthcare systems. However, data on the use of these therapies among oncology physicians and patients in Southeast Europe remains limited. This study aims to evaluate the accuracy of oncologists’ decisions regarding whether specific complementary therapies should be contraindicated, permitted, or recommended in early BC (eBC), utilizing a vignette-based approach grounded in existing literature.

## Materials and methods

### Study design

This cross-sectional online survey employed a randomized vignette experiment. Respondents were randomly assigned to one of eight patient profiles, defined by a 2×2×2 factorial combination of patient age (35 vs 75 years), education level (primary vs university), and nodal status (N0 vs N1). The allocation across the eight profiles is detailed in Table S1. Each participant assessed 56 therapy-indication decisions (28 complementary therapy options, evaluated once in the neoadjuvant (NA) and once in the adjuvant (ADJ) setting) for the assigned patient profile. This analysis forms part of a broader study investigating oncologists’ use and perceptions of complementary and alternative medicine (CAM) in eBC. The current analysis specifically focuses on oncologists’ evidence-based decision-making regarding complementary therapies and excludes alternative therapies. While the study protocol was not preregistered on a public registry, it was submitted as a proposal for the first author’s doctoral thesis and is publicly accessible in the University of Mostar Faculty of Medicine repository.

### Participants

Eligible participants comprised oncology specialists and residents who actively treated women with BC at least once a month. Local collaborators from seven countries—Bosnia and Herzegovina, Bulgaria, Croatia, Montenegro, North Macedonia, Serbia, and Slovenia—distributed invitations and links to the survey. While the LimeSurvey platform included Hungary and Romania as options, only responses from the targeted Southeast European countries (Bosnia and Herzegovina, Bulgaria, Croatia, Montenegro, North Macedonia, Serbia, and Slovenia) were included in the analytical sample. The questionnaire was distributed electronically via the LimeSurvey platform, utilizing a convenience sampling method. Participation was voluntary, and responses were collected anonymously. To prevent duplicate submissions, multiple responses from the same access code were restricted. The survey was accessible through a single, open link compatible with standard web browsers and devices. Before data collection, we conducted an *a priori* power analysis using PASS 2021 for a clustered design, with country as the sole random source of variation. We assumed an intraclass correlation coefficient (ICC) of 0.05, an alpha level of 0.05, and 80% power to detect a standardized mean difference of Cohen’s *d* ═ 0.50, which indicated a target of approximately 257 respondents. The actual analytical sample consisted of 136 respondents across seven countries, resulting in a design effect of approximately 2.4 (effective *n* ≈ 56), providing sufficient power only for large adjusted effects (approximately *d* ≈ 0.75; around 10 percentage points under strict definitions and approximately 7–8 percentage points under expanded accuracy definitions).

### Outcomes

The study employed clinical vignettes in which a woman with eBC receiving chemotherapy inquired about using a specific complementary therapy for a defined indication. For each vignette, participants indicated whether they would prevent, allow, or recommend the use of the suggested complementary therapies across seven clinical domains: peripheral neuropathy, fatigue, nausea/vomiting, anemia, mood disorders, sleep disturbances, and quality of life. Scoring keys were established *a priori* based on evidence-based guidelines for integrative oncology, primarily referencing Greenlee et al. (2017) and Witt and Cardoso (2016), which evaluate the efficacy and safety of complementary therapy modalities by indication [5, 6]. The prespecified scoring keys were reviewed against the 2023–2024 consensus updates for fatigue and anxiety/depression [31–33]. Concordance was confirmed, and no re-analysis was necessary. In instances where the sources did not address a specific therapy-indication pair, contemporary peer-reviewed literature was utilized to inform the scoring [12, 34–43]. Therapies with moderate or strong evidence for the indication were classified as “recommend”; those with limited or uncertain benefit but acceptable safety were classified as “allow”; and therapies demonstrating harm, poor tolerability, or significant interaction risk were classified as “prevent.” Participants were not provided with evidence ratings.

The primary outcome measured the correctness of evidence-aligned decisions regarding complementary therapies across NA and ADJ settings, defined as the proportion of correctly classified therapy–indication pairs out of 28 in each setting (56 overall). Two related measures of correctness were prespecified. The strict correctness rule served as the primary definition, counting only the single evidence-concordant action as correct. The expanded correctness rule, treated as a prespecified secondary measure, encompassed a broader construct where responses aligned directionally with the evidence but not fully concordant with the strict rule were also considered correct.

Secondary outcomes included: (i) item-level distributions of responses (prevent/allow/recommend) pooled across settings, and (ii) item-level endorsement rates (allow + recommend).

The outcome measures were developed through a literature review, expert discussions, and a pilot study involving 12 oncologists and oncology residents, followed by revisions for clarity. They were not validated on an independent sample, and their psychometric properties were unknown in advance. The item set and coding scheme were not designed as a psychometric scale and were analyzed as decision classifications rather than latent scores.

### Ethical statement

This study was conducted in accordance with the Declaration of Helsinki. Ethical approval was obtained from the Ethics Committee of the University of Mostar, Faculty of Medicine (Approval No. 01-I-561/24). Participation was voluntary, and completion of the anonymous online survey constituted informed consent.

### Statistical analysis

Prior to database lock, we finalized and documented the analytical framework, which included defining the primary endpoint, covariates, and planned sensitivity analyses in the doctoral project protocol. We adhered to this prespecified plan without any post-hoc analytical modifications following data inspection. Among the 136 participants included in the primary analysis (valid paired responses across both NA and ADJ settings), three item-level responses were missing out of a total of 7616 possible responses (28 items × 2 settings × 136 participants), resulting in a missingness rate of 0.039%. To maintain paired comparisons, we employed a deterministic single-value imputation method: when a respondent exhibited perfect internal consistency within a setting, we imputed the within-respondent modal response; otherwise, we utilized the cohort-level modal response for that item. For descriptive purposes, denominators reflect the actual number of responses per item, with a maximum of 272 responses per item. Three items (Qi Gong, Comfrey, PC-SPES) had *n* ═ 271 due to one missing paired response each. Given the negligible missingness, multiple imputation was deemed unnecessary.

The primary outcome assessed participant-level accuracy across 56 therapy-indication decisions. We summarized medians and interquartile ranges (IQRs), while 95% confidence intervals (CIs) for medians were calculated using the Bonett–Price method [44]. Accuracy was computed per participant by evidence category, restricted to items within that category, and then summarized accordingly. 95% CIs for participant-level proportions were derived using logit-transformed binomial intervals, treating each participant as the unit of analysis.

In a sensitivity analysis, we reweighted each respondent’s NA responses according to their self-reported annual percentage of NA patients, while assigning unit weight to ADJ responses (as all respondents routinely treat patients in the ADJ setting). Practice-weighted accuracy was defined as (accADJ × 1 + accNA × propNA) / (1 + propNA), where accADJ represents accuracy of decisions in the ADJ setting, accNA indicates accuracy of decisions in the (NA setting), and propNA is the proportion of patients treated with NA therapy by each participant.

In an additional sensitivity analysis, we weighted accuracy by the absolute number of patients treated in the NA setting to approximate real-world clinical impact. The annualized number of NA patients (2 × the number reported for six months) and the annual number of BC patients (12 × monthly caseload) were used to estimate NA and ADJ volumes, respectively. Impact-weighted accuracy per oncologist was defined as (accADJ × ADJvolume + accNA × NAvolume) / (ADJvolume + NAvolume), where ADJvolume and NAvolume represent the estimated number of patients treated annually in each respective setting. We also reported a population-level impact-weighted median across respondents using these volumes as analytical weights. Weighted and unweighted distributions were compared using the Wilcoxon signed-rank test to evaluate potential differences between practice-weighted and unweighted estimates.

Considering the disparities between the country distribution in our sample and the underlying regional oncology workforce, we conducted a post-stratification sensitivity analysis using country weights (population share/samples share). Weighted medians and IQRs were obtained using weighted quantiles, and 95% CIs for medians were computed through bootstrapping (2000 replicates). For clarity, non-parametric bootstrap 95% CIs (2000 replicates) were calculated and reported exclusively for the country-weighted medians. Practice-weighted and impact-weighted medians were presented as descriptive robustness summaries without CIs and were compared to the corresponding unweighted medians using the Wilcoxon signed-rank test. Estimates of the oncology workforce size in each country were derived from a key-informant survey conducted in June 2015 among senior oncologists in Bosnia and Herzegovina, Bulgaria, Croatia, Montenegro, North Macedonia, Serbia, and Slovenia, which was initially designed for earlier research on cancer control in the region [45]. Where feasible, these estimates were cross-verified with senior representatives of pharmaceutical companies active in oncology within the respective countries to ensure they accurately reflect clinicians routinely involved in systemic anticancer therapy.

We modeled participant-level accuracy using beta regression with a logit link and Huber-White robust standard errors. In instances where boundary values (0 or 1) occurred, we applied the Smithson–Verkuilen transformation [46]. Primary models incorporated randomized vignette attributes (age, education, nodal status) and country fixed effects. “Full” models additionally included clinician characteristics (gender, professional status, specialty, years in oncology, institution type, number of BC patients treated monthly, frequency of patient contacts, number of patients treated with NA therapy, and their share of the total patient population). We report predictive margins (Acc) and average marginal effects (AME) with delta-method 95% CIs derived from margins. For categorical predictors, Acc reflects the predictive margin for each level, while AME represents the contrast against the reference level. For continuous predictors, Acc is the predictive margin evaluated at the sample median, and AME indicates the change per +10 units. We fitted all models using the strict correctness rule as the primary definition of correctness and subsequently re-estimated them using the expanded correctness rule to illustrate the extent to which substantive conclusions depended on the choice of a narrower vs a broader correctness construct. For the multivariable beta-regression models, we controlled for the false discovery rate (FDR) using the Benjamini–Hochberg procedure with an FDR set at < 5%.

**Figure 1.**
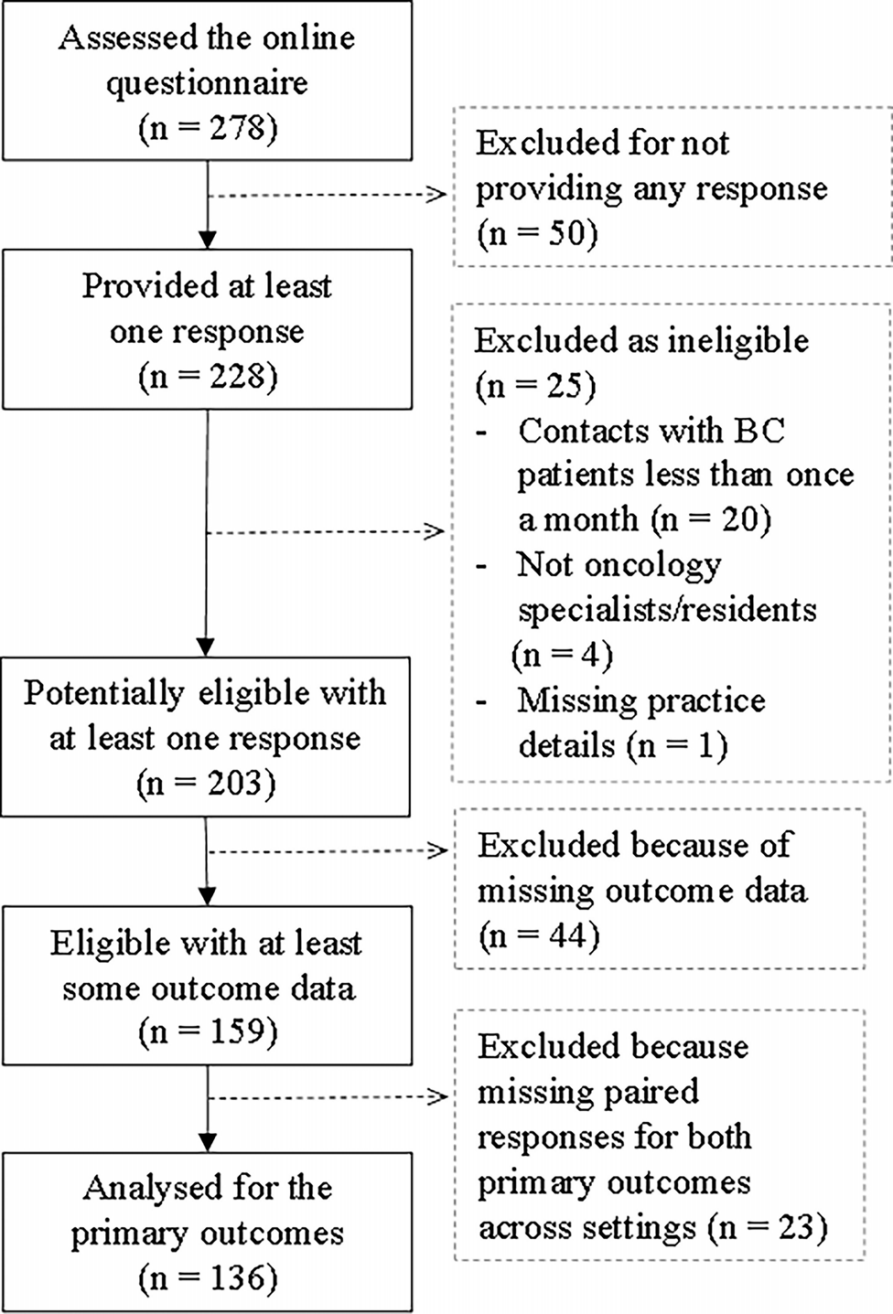
**Study sample and participant flow.** Flow of participants from initial survey access to inclusion in the final analysis (*n* ═ 136 valid paired responses).

**Table 1:** Participants characteristics; unweighted data (*n* ═ 136)

	***n* (%)**
Country	
Bosnia and Herzegovina	33 (24.3)
Bulgaria	10 (7.4)
Croatia	48 (35.3)
Montenegro	7 (5.1)
North Macedonia	11 (8.1)
Serbia	17 (12.5)
Slovenia	10 (7.4)
Age (years), median [IQR]	38 [34–45]
Gender	
Men	39 (28.7)
Women	97 (71.3)
Professional status	
Residents	39 (28.7)
Specialists	97 (71.3)
Specialization	
Medical oncologist	84 (61.8)
Clinical oncologist	40 (29.4)
Radiation oncologist	12 (8.8)
Work experience in oncology (years), median [IQR]	10 [5–15]
Type of institution	
University hospital	89 (65.4)
General hospital	34 (25.0)
Spec. oncology institution	13 (9.6)
Number of BC patients monthly, median [IQR]	40 [20–88]
Frequency of contacts with BC patients	
Daily	98 (72.1)
Weekly or less often	38 (27.9)
Number of BC patients treated with NA CHT during six months, median [IQR]	20 [10–39]
Percentage of patients with eBC treated NA annually, median [IQR]	30 [20–58]

Data are presented as a percentage of participants unless otherwise specified. Abbreviations: IQR: Interquartile range; BC: Breast cancer; eBC: Early breast cancer; NA: Neoadjuvant; CHT: Chemotherapy.

**Table 2:** Accuracy of evidence-aligned decisions concerning complementary therapies, pooled from neoadjuvant and adjuvant settings (*n* ═ 136; *m* ═ 56 therapy-indication pairs per participant)

	**Median [IQR]**	**(95% CI)**	**Min.**	**Max.**
Strict definition of correctness	52 [43–63]	(48–55)	21	82
Expanded definition correctness	70 [61–76]	(67–72)	38	93
*By evidence category*				
Supported for use (allow or recommend) (*m* ═ 30)	80 [70–90]	(77–83)	3	100
Supported for recommendation (*m* ═ 14)	50 [14–82]	(39–61)	0	100
Supported to be allowed but not recommended (*m* ═ 16)	69 [50–88]	(62–75)	0	100
Evidence advising against use (to be prevented) (*m* ═ 26)	60 [37–77]	(54–65)	0	100

The strict rule recognizes only the single evidence-concordant action as correct, while the expanded rule permits or recommends actions based on supporting evidence. The variable *m* represents the number of therapy-indication pairs evaluated across both clinical settings. Seven indications were associated with four complementary therapies, resulting in 28 therapy-indication combinations assessed separately in adjuvant and neoadjuvant contexts, yielding a total of 56 evaluations. The categories “supported for recommendation” (*m* ═ 14) and “supported to be allowed” (*m* ═ 16) collectively form the category “supported for use” (*m* ═ 30). Abbreviations: M: Number of complementary therapies evaluated; IQR: Interquartile range; CI: Bonett–Price confidence interval for the median; Min.: Minimum; Max.: Maximum.

Item-level differences between NA and ADJ settings were analyzed using paired McNemar tests, with Cohen's g as the standardized effect size. These analyses were not prespecified and were treated as exploratory. Consequently, we report raw (unadjusted) *P* values without correction for multiple comparisons and interpret them descriptively. We do not regard these exploratory *P* values as evidence of statistical significance or non-significance. In our questionnaire, all NA items were presented as a block before all ADJ items for every respondent, and the order of NA and ADJ blocks was not randomized. All tests were two-sided. Statistical analysis was conducted using StataCorp 2019 (Stata Statistical Software: Release 16, College Station, TX, USA: StataCorp LLC).

## Results

### Participant characteristics

Between July 13, 2024, and May 3, 2025, 278 individuals accessed the online questionnaire, resulting in a final sample of 136 participants with valid paired responses (Figure 1). The last response was received on May 3, 2025, and the survey was officially closed on May 13, 2025, at 23:51 Central European Time, as indicated by the system timestamp on the printable questionnaire (Supplementary File 1). Participants were predominantly female, with most being medical oncologists practicing in university hospitals. The median age was 38 years (IQR 34–45), and the median years of oncology experience was 10 (IQR 5–15) (Table 1). The majority reported daily contact with BC patients, with a median of 40 patients examined monthly (IQR 20–88). In the previous six months, the median number treated with NA chemotherapy was 20 (IQR 10–39). Participants were recruited from seven Southeast European countries with unequal contributions. Between-country comparisons were not planned, and the study was not powered for such analyses. The distribution of respondents by country did not fully reflect the regional oncologist population structure, with some countries overrepresented and others underrepresented in the sample (Table S2).

### Primary outcome

Across 56 therapy-indication decisions per participant, the median accuracy was 52% (95% CI 48–55) under the strict definition of correctness and 70% (95% CI 67–72) under the expanded definition (Table 2). By evidence category, accuracy was 80% (95% CI 77–83) for therapies supported for use (allow or recommend), 50% (95% CI 39–61) for those supported for recommendation, 69% (95% CI 62–75) for therapies supported to be allowed but not recommended, and 60% (95% CI 54–65) for therapies for which evidence advises against use.

Practice-weighted accuracy estimates, calculated as a sensitivity analysis, were nearly identical to the primary balanced analysis (Wilcoxon signed-rank test; strict definition: median 52% vs 52%, unadjusted *P* ═ 0.669; expanded definition: median 68% vs 70%, unadjusted *P* ═ 0.279). This indicates that accounting for variability in the proportion of NA patients treated by each oncologist did not impact the results.

Impact-weighted accuracy was similarly aligned with the balanced analysis (Wilcoxon signed-rank test; strict definition: median 51% vs 52%, unadjusted *P* ═ 0.768; expanded definition: median 68% vs 70%, unadjusted *P* ═ 0.281). At the population level, impact-weighted mean accuracy was 53% (strict) and 68% (expanded), closely mirroring balanced estimates. These findings confirm the robustness of the main results across different weighting schemes.

Country-weighted (post-stratified) sensitivity analysis, which reweighted respondents to match the national composition of the regional oncology workforce (weights in Table S2), yielded medians of 54% (IQR 43–64; bootstrap 95% CI 49–58) for the strict definition and 68% (IQR 61–75; bootstrap 95% CI 64–72) for the expanded definition—values similar to the unweighted estimates. Collectively, these sensitivity analyses affirm the robustness of the main findings against alternative weighting schemes.

### Secondary outcomes

**Figure 2.**
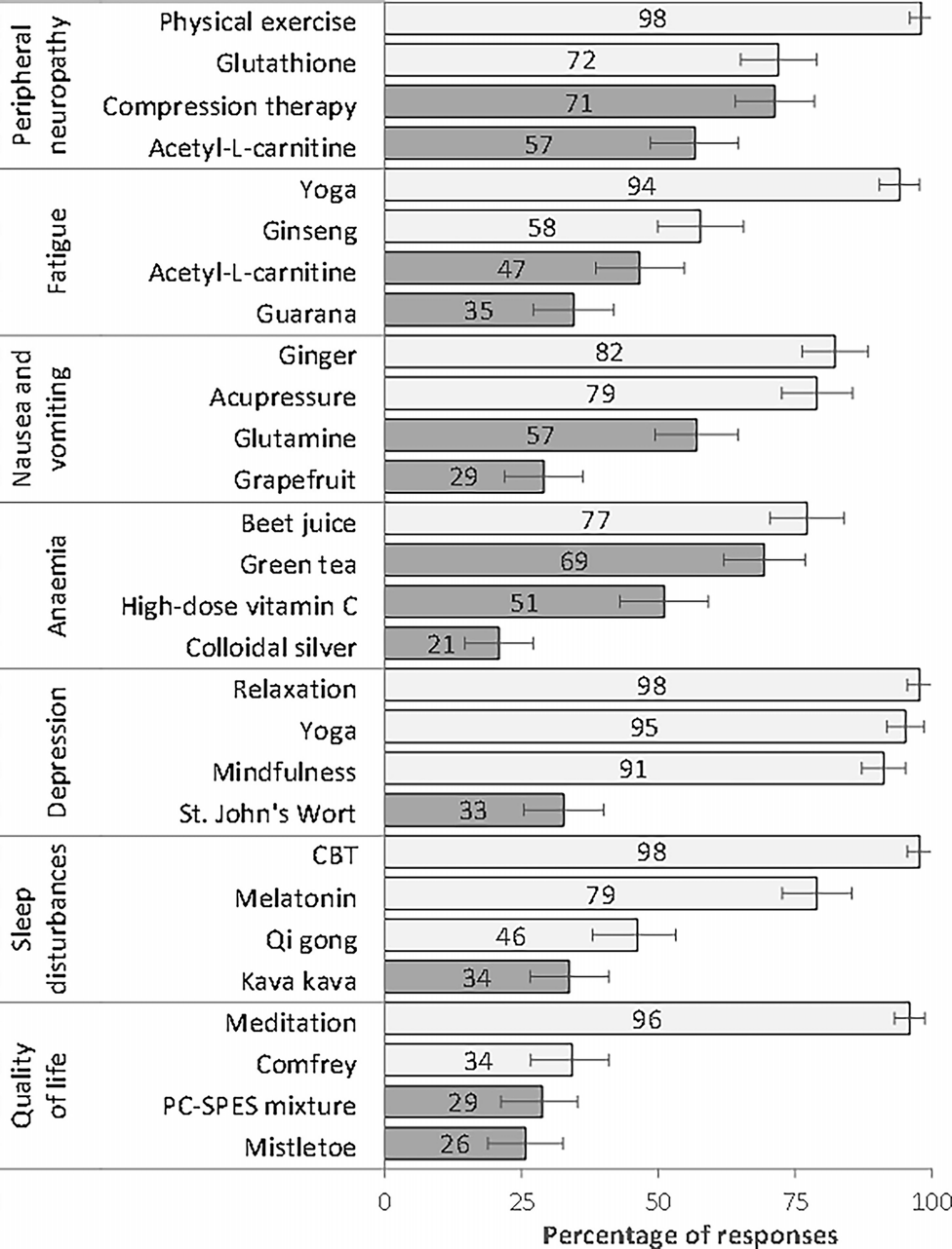
**Endorsement of complementary therapies in neoadjuvant and adjuvant settings.** The bars represent the percentage of responses categorized by endorsement for each indication. Error bars illustrate the 95% confidence intervals for participant-level proportions, calculated using logit-transformed binomial intervals (*n* ═ 136; each participant contributed two vignette responses; unweighted data). Dark bars indicate therapies for which existing evidence recommends against use (“prevent”), reflecting responses that are not aligned with the evidence.

Endorsement (allow + recommend) varied significantly across items (Figure 2; Table 3). The highest rates were observed for evidence-supported options: physical exercise for neuropathy and cognitive behavioral therapy for sleep (both ≈ 98%). Conversely, several therapies advised against were still frequently endorsed, including glutathione for neuropathy and green tea for anemia (≈ 72% and ≈ 69%). Items supported only to be allowed showed mixed uptake (e.g., ginseng for fatigue ≈ 58%, qi gong for sleep ≈ 46%, mistletoe for quality of life ≈ 26%) (Table S3).

Contrasts between NA and ADJ settings were not a prespecified outcome and were analyzed exploratorily. Overall, these comparisons revealed no meaningful differences. The only items with nominally significant differences between settings were comfrey for quality of life (McNemar test; unadjusted *P* ═ 0.019; Cohen’s *g* ═ –0.09) and acupressure for nausea/vomiting (McNemar test; unadjusted *P* ═ 0.035; Cohen’s *g* ═ 0.05), with otherwise uniformly trivial effect sizes (Tables S4 and S5).

In multivariable beta-regression models with country fixed effects, adjusted simultaneously for all vignette and clinician covariates, accuracy differed by specialty and frequency of contact with BC patients (Table 4). Compared to medical oncologists, clinical oncologists exhibited lower accuracy under both definitions (strict AME −5.3 pp, 95% CI −10.4 to −0.2; *P* ═ 0.043; expanded AME −4.6 pp, −9.0 to −0.2; *P* ═ 0.040), but in both cases, the FDR was >5%. Radiation oncologists demonstrated greater deficits (strict −8.0 pp, −13.2 to −2.9; *P* ═ 0.002; FDR < 5%; expanded −10.3 pp, −15.1 to −5.5; *P* < 0.001; FDR < 5%). Seeing BC patients weekly or less often (vs daily) was associated with lower accuracy under the expanded definition (−4.5 pp, −8.1 to −0.9; *P* ═ 0.013; FDR < 5%). No other vignette attributes (age, education, nodal status) or clinician/institutional measures (gender, professional status, experience, institution type, number of BC patients monthly, number or share of patients treated with NA therapy) reached statistical significance with FDR < 5%.

## Discussion

The results of our study indicate a moderate alignment among Southeast European oncologists regarding their decisions and the best available evidence on the efficacy and safety of complementary therapies in eBC. Under a strict definition of correctness, participants accurately identified approximately half of the complementary therapy-indication pairs consistent with published evidence. However, when both “recommend” and “allow” were considered acceptable in contexts where evidence supported either approach, the median accuracy increased to 70%. Among complementary therapies backed by clinical evidence, participants achieved a median accuracy of 80%, with a higher recognition rate for therapies deemed permissible compared to those explicitly recommended. This pattern suggests a prevailing caution among oncologists, who appear more willing to tolerate complementary therapies than to endorse them outright, even in the presence of robust evidence supporting their benefits and safety. Sensitivity analyses confirmed the robustness of these findings, indicating that neither the individual clinical focus of the oncologists nor the number of patients treated significantly influenced the observed accuracy.

Studies from Ireland and Jordan have reported limited knowledge about complementary therapies among healthcare professionals [47, 48]. A multicenter study in Italy examining physicians’ attitudes toward complementary therapies found oncologists to be the most informed group [49]. In the United States, a national survey revealed that two out of three oncologists felt insufficiently knowledgeable to address patients’ inquiries regarding complementary therapies [50]. In China, approximately one-third of oncologists had discussed complementary therapies with their patients, with most not initiating such conversations. Four out of five oncologists expressed a lack of knowledge and did not support the use of complementary therapies by their patients [51]. These studies consistently highlight insufficient knowledge, limited training, and a lack of confidence among healthcare professionals in guiding patients. Unlike previous studies that relied on self-reported assessments of perceived knowledge, our research employed a case-based, evidence-grounded approach to evaluate oncologists’ ability to apply clinical guidelines in realistic decision-making scenarios. This methodological distinction is significant, as self-perception may not accurately reflect the ability to make evidence-concordant decisions in practice. The vignette-based method offers clearer insights into the gap between perceived competence and actual performance, which is critical for developing targeted educational interventions. By utilizing clinical vignettes and comparing responses against established guidelines and published safety data, our study provides a more objective assessment of decision accuracy. To our knowledge, this is the first study in Southeast Europe to evaluate oncologists’ evidence-based understanding of complementary therapies in oncology using a structured and standardized scoring system.

Our findings suggest that the threshold for explicitly recommending complementary treatments, as opposed to merely allowing them, remains relatively high among oncology professionals. Even for treatments supported by moderate or strong evidence, many respondents opted to allow rather than recommend them. This behavior likely reflects a cautious clinical approach, influenced by concerns about patient expectations, therapeutic responsibility, or the strength of the supporting evidence. The minimal differences in recommendation patterns between NA and ADJ settings imply that clinical context does not substantially alter oncologists’ attitudes toward these therapies. However, the low overall recommendation rates for most treatments—especially those with limited or questionable efficacy—underscore the ongoing tension between openness to integrative approaches and adherence to evidence-based practice. These patterns emphasize the necessity for continued professional education and clear communication regarding the evidence base for complementary interventions in oncology.

The present findings reveal significant inconsistencies in oncologists’ ability to align their decisions regarding complementary therapies with published evidence. Although overall accuracy improved when a more inclusive definition of correctness was applied, a substantial proportion of responses remained incongruent with evidence-based guidance [5, 6]. Therapies supported by evidence for either allowance or recommendation were generally identified with higher accuracy than those for which use is advised against. However, a nuanced pattern emerged when disaggregating by response type: therapies supported for recommendation were associated with the lowest accuracy, despite representing the strongest form of endorsement. In contrast, accuracy for therapies deemed permissible (but not recommended) was higher, though not significantly different from that for therapies with evidence discouraging use. These findings suggest that participants may hesitate to actively recommend complementary therapies, even when robust evidence is available, possibly reflecting caution, perceived professional risk, or limited familiarity with the evidence base. Moreover, the expanded definition of correctness—accepting either allow or recommend when evidence supports either option—may overestimate actual alignment, as it provides two acceptable options for therapies supported for use but only one for those discouraged. This asymmetry should be considered when interpreting comparative accuracy levels. Such patterns reflect previous studies indicating that oncologists are generally receptive to complementary therapies perceived as safe, even in the absence of strong efficacy data, but are less consistent in recognizing those contraindicated due to harm, ineffectiveness, or interactions with conventional treatments [5, 6]. These gaps may indicate a tension between professional openness to patient-centered, integrative approaches and the demands of rigorous evidence-based practice. From an educational perspective, the findings underscore the need for targeted training in the evaluation and application of evidence concerning complementary therapies.

From our clinical experience, oncologists in Southeast Europe rarely initiate discussions about complementary therapies with patients, despite their relevance, due to a perceived lack of information. Patients in this region frequently inquire about herbal medicines and dietary supplements, which may interact with oncology treatments and necessitate careful consideration. Consequently, oncologists might find it easier to allow patients to pursue these therapies independently, thereby transferring responsibility to the patient. In contrast, mind–body interventions such as mindfulness, relaxation techniques, and yoga are less commonly requested and recommended, as they are not widely adopted in Southeast Europe compared to Western countries. However, given their low risk of harm or interactions, clinicians may feel more comfortable permitting or recommending these interventions. This cautious approach is further exacerbated by the relatively underdeveloped state of integrative medicine in the region, underscoring the need for region-specific educational resources and confidence-building tools to support the evidence-based integration of complementary interventions into routine care.

**Table 3:** Willingness to prevent, allow, or recommend complementary therapies in neoadjuvant and adjuvant settings

**Indication**	**“Correct”**	**Pooled actual responses**		
**Treatment**	**response**	**prevent**	**allow**	**recommend**
Prevention of peripheral neuropathy				
Physical exercise	allow	5 (1.8)	58 (21.3)	209 (76.8)
Compression therapy	allow	78 (28.7)	152 (55.9)	42 (15.4)
Glutathione	prevent	76 (27.9)	169 (62.1)	27 (9.9)
Acetyl-L-carnitine	prevent	118 (43.4)	134 (49.3)	20 (7.4)
Fatigue				
Yoga	recommend	16 (5.9)	112 (41.2)	144 (52.9)
Ginseng	allow	115 (42.3)	136 (50.0)	21 (7.7)
Guarana	prevent	178 (65.4)	87 (32.0)	7 (2.6)
Acetyl-L-carnitine	prevent	145 (53.3)	116 (42.6)	11 (4.0)
Nausea and vomiting				
Acupressure	recommend	57 (21.0)	157 (57.7)	58 (21.3)
Ginger	allow	48 (17.6)	166 (61.0)	58 (21.3)
Grapefruit	prevent	193 (71.0)	72 (26.5)	7 (2.6)
Glutamine	prevent	117 (43.0)	140 (51.5)	15 (5.5)
Anemia				
Beet juice	allow	62 (22.8)	173 (63.6)	37 (13.6)
Colloidal silver	prevent	215 (79.0)	52 (19.1)	5 (1.8)
High-dose vitamin C	prevent	133 (48.9)	122 (44.9)	17 (6.2)
Green tea	prevent	83 (30.5)	178 (65.4)	11 (4.0)
Depression and other mood disorders				
Mindfulness	recommend	24 (8.8)	121 (44.5)	127 (46.7)
Yoga	recommend	13 (4.8)	132 (48.5)	127 (46.7)
Relaxation	recommend	6 (2.2)	78 (28.7)	188 (69.1)
St. John’s Wort	prevent	183 (67.3)	81 (29.8)	8 (2.9)
Sleep disturbances				
CBT	recommend	6 (2.2)	99 (36.4)	167 (61.4)
Melatonin	allow	57 (21.0)	182 (66.9)	33 (12.1)
Qi gong	allow	146 (53.9)	108 (39.9)	17 (6.3)
Kava kava (lat. *Piper methysticum*)	prevent	180 (66.2)	86 (31.6)	6 (2.2)
Quality of life				
Meditation	recommend	11 (4.0)	119 (43.8)	142 (52.2)
Mistletoe	allow	202 (74.3)	66 (24.3)	4 (1.5)
Comfrey	prevent	178 (65.7)	89 (32.8)	4 (1.5)
PC-SPES mixture	prevent	193 (71.2)	75 (27.7)	3 (1.1)

Values represent the number (percentage) of responses, not the number of participants (denominator per item = 272; each participant provided responses for two vignette settings). Three items have *n* ═ 271 due to one missing paired response for each (Qi Gong, Comfrey, PC-SPES). Data are presented as frequency (percentage) of responses rather than participants. The “Correct Response” column identifies the evidence-based action corresponding to each therapy-indication pair, as outlined in published guidelines (Greenlee et al., 2017; Witt and Cardoso, 2016). Respondents were not provided with evidence ratings while completing the survey; therefore, percentages may not total 100 due to rounding. Abbreviations: CBT: Cognitive behavioural therapy; PC-SPES: Prostate Cancer – Special Extract Series (herbal mixture).

**Table 4:** Accuracy and adjusted effects (AME, percentage points) of vignette attributes and clinician characteristics under strict and expanded accuracy definitions (*n* ═ 136)

		**Strict definition**				**Expanded definition**			
	***n***	**Acc.**	**AME**	**(95% CI)**	***P***	**Acc.**	**AME**	**(95% CI)**	***P***
*Patient attributes form vignettes*									
Age									
35 years	59	52.0	Ref.			69.3	0.0		
75 years	77	52.6	0.6	(−3.7 to 5.0)	0.776	68.2	−1.1	(−4.6 to 2.4)	0.541
Education									
Primary school	72	53.2	Ref.		.	69.3	0.0		.
University	64	51.4	−1.8	(−5.8 to 2.1)	0.360	68.0	−1.3	(−4.3 to 1.7)	0.405
Lymph nodes									
Negative	69	53.6	Ref.		.	70.2	0.0		.
Positive	67	51.1	−2.5	(−6.8 to 1.8)	0.251	67.1	−3.0	(−6.6 to 0.5)	0.093
*Clinicians’ (respondents) characteristics*									
Gender									
Men	39	50.1	Ref.		.	66.9	0.0		.
Women	97	53.3	3.2	(−1.5 to 7.9)	0.186	69.4	2.6	(−1.1 to 6.2)	0.167
Professional status									
Residents	39	52.1	Ref.		.	67.3	0.0		.
Specialists	97	52.5	0.3	(−5.3 to 6.0)	0.905	69.2	1.9	(−2.7 to 6.4)	0.420
Specialization									
Medical oncologist	84	54.6	Ref.		.	70.9	0.0		.
Clinical oncologist	40	49.3	−5.3	(−10.4 to −0.2)	0.043	66.3	−4.6	(−9.0 to −0.2)	0.040
Radiation oncologist	12	46.6	−8.0	(−13.2 to −2.9)	0.002*	60.6	−10.3	(−15.1 to −5.5)	0.000*
Work experience in oncology	136	52.6	−1.5	(−4.8 to 1.8)	0.379	68.8	−0.7	(−3.7 to 2.3)	0.658
Type of institution									
University hospital	89	53.3	Ref.		.	69.4	0.0		.
General hospital	34	50.9	−2.5	(−8.3 to 3.3)	0.400	67.1	−2.3	(−7.2 to 2.6)	0.360
Spec. oncology institution	13	49.7	−3.7	(−12.0 to 4.7)	0.389	68.2	−1.2	(−6.8 to 4.5)	0.691
Number of BC patients monthly	136	52.8	−0.1	(−0.3 to 0.1)	0.361	69.2	−0.1	(−0.3 to 0.1)	0.164
Frequency of contacts with BC patients									
Daily	98	53.5	Ref.		.	69.9	0.0		.
Weekly or less often	38	49.4	−4.2	(−8.7 to 0.4)	0.071	65.4	−4.5	(−8.1 to −0.9)	0.013*
Number of BC patients treated with NA CHT during six months	136	51.9	0.1	(−0.0 to 0.3)	0.146	68.5	0.1	(−0.1 to 0.2)	0.358
Percentage of patients with eBC treated NA annually	136	52.7	−0.4	(−1.4 to 0.7)	0.518	69.1	−0.5	(−1.4 to 0.4)	0.248

Note: *FDR < 5%. The accuracy (Acc.) values presented are predictive margins derived from a beta-regression model utilizing a logit link, incorporating country fixed effects, robust standard errors, and the Smithson–Verkuilen transformation where applicable. For categorical predictors, Acc. denotes the predictive margin for each level; for continuous predictors, it reflects the predictive margin with the predictor set to its sample median, while averaging all covariates over their observed distribution. Average marginal effects (AME) are expressed in percentage points: for categorical predictors, they indicate the difference compared to the reference level (Ref.); for continuous predictors, they represent the change associated with an increase of 10 units in the predictor (a negative AME suggests lower accuracy relative to the reference). Abbreviations: *n*: Number of respondents in each row; Acc.: Predicted accuracy (%); AME: Average marginal effect (percentage points); 95% CI: 95% confidence interval; *P*: *P* value; Ref.: Reference category; BC: Breast cancer; eBC: Early breast cancer; NA: Neoadjuvant; CHT: Chemotherapy.

While vignette-based assessments provide a standardized measure of decision-making accuracy, they cannot fully capture the complexities of real-world encounters, including patient preferences, institutional constraints, and multidisciplinary input. These factors may influence oncologists’ recommendations, suggesting that observed patterns of caution may either underestimate or overestimate actual clinical behavior.

Another significant challenge is the persistent lack of terminological clarity surrounding complementary, alternative, and integrative therapies. Although conceptually distinct, their definitions vary across professional societies, patient-facing organizations, and research publications. For instance, complementary therapies are typically described as adjuncts to conventional oncology treatment, while alternative therapies are positioned as replacements for evidence-based care; integrative approaches refer to the coordinated use of supportive modalities within conventional oncology. Despite these distinctions, the umbrella term CAM is often applied inconsistently, even in studies focused solely on complementary use. From a clinician’s perspective, this conceptual ambiguity complicates communication, contributes to variable interpretation of evidence, and may influence clinical decision-making. Addressing this lack of standardization is essential for enhancing both research comparability and real-world application of supportive therapies in oncology.

### Limitations of the study

This study has several important limitations. First, we utilized a non-random, convenience sample with unknown representativeness for the targeted population, likely over-representing clinicians with a particular interest in complementary therapies. Second, the sample size was modest and fell short of the *a priori* target, which reduced statistical power and limited the precision and stability of subgroup-specific estimates. Third, the scoring system, although grounded in current evidence and guidelines, required interpretation in cases where recommendations were not explicitly stated or where evidence was evolving. While this approach was carefully designed and pilot-tested, the classification of certain responses as correct or incorrect may remain debatable. Fourth, the cross-sectional, vignette-based design assessed decision-making accuracy in hypothetical but standardized clinical contexts rather than in observed clinical practice, which may limit ecological validity and precludes assessment of longitudinal changes in clinician knowledge or behavior. Fifth, we did not include dedicated manipulation-check items (for example, ratings of perceived prognosis or risk), so we cannot empirically verify the extent to which respondents perceived the intended differences between patient profiles. We therefore interpret any apparent influence of patient attributes on decisions cautiously, maintaining our primary focus on correctness relative to trial evidence and guideline-based standards of care. Sixth, the study evaluates oncologists’ decision-making accuracy but does not assess downstream patient outcomes such as survival, adherence, or disease progression, making conclusions about clinical impact indirect. Seventh, in the post-stratification sensitivity analysis, we applied country weights (population share/sample share). Target population sizes in different countries were based on key-informant estimates from 2015, which may limit validity, reliability, and precision. Our workforce estimates diverge from figures reported in other surveys and more recent data sources [52, 53]. Eighth, in the questionnaire, all NA items were presented before all ADJ items, so the effect of clinical setting (NA vs ADJ) is intrinsically confounded with block order. Thus, we cannot exclude the possibility that some observed NA–ADJ differences reflect order or fatigue effects rather than true setting-specific behavior.

Given the increasing patient demand for integrative oncology, consistent and well-informed guidance from oncologists is essential. Decision aids, concise evidence summaries tailored to clinical settings, and the inclusion of complementary therapies in continuing medical education may help reduce variability and misalignment. Although this study did not assess clinical outcomes, it raises important concerns regarding shared decision-making and the accuracy of information communicated to patients. Future research should examine whether discrepancies between clinician decisions and evidence contribute to overuse, underuse, or miscommunication regarding complementary therapies, and whether targeted interventions can enhance the fidelity of evidence-based decisions in oncology.

## Conclusion

This study provides insight into decision patterns regarding complementary therapies among surveyed oncologists in Southern Europe, though findings should be interpreted cautiously due to non-probability sampling and limited representativeness. While moderate familiarity with evidence-based complementary therapies was observed, significant gaps and inconsistencies remain, particularly concerning the active recommendation of therapies with proven benefits. Bridging these gaps through targeted education and the integration of clinical decision support tools is essential to ensure the safe, evidence-based incorporation of complementary therapies into oncology care.

We recommend:

- Developing concise, evidence-based educational materials on complementary therapies for oncologists.
- Including complementary therapies guideline content in national oncology training programs across Southeast Europe.
- Providing institutional support mechanisms, such as complementary therapies counselors, to assist oncologists in navigating patient queries.

By addressing these gaps, we can promote safer and more integrated oncology practices, ultimately enhancing patient care and outcomes.

## Supplemental data

Supplemental data are available at the following links: https://www.bjbms.org/ojs/index.php/bjbms/article/view/13413/4081. https://www.bjbms.org/ojs/index.php/bjbms/article/view/13413/4097.

## Data Availability

The questionnaire used in this study is provided as Supplementary File 1 (PDF). Other datasets supporting the conclusions of this article are included within the article and its supplementary materials or are available from the corresponding author upon reasonable request.
